# A systematic review and network meta-analysis of virtual reality, audiovisuals and music interventions for reducing dental anxiety related to tooth extraction

**DOI:** 10.1186/s12903-023-03407-y

**Published:** 2023-09-22

**Authors:** Tiedan Hao, Jun Pang, Qingmei Liu, Pengfei Xin

**Affiliations:** 1 Department of Operating Room, Shanxi Bethune Hospital, Shanxi Academy of Medical Sciences, Tongji Shanxi Hospital, Third Hospital of Shanxi Medical University, Taiyuan, 030032 China; 2 Tongji Hospital, Tongji Medical College, Huazhong University of Science and Technology, Wuhan, 430030 China; 3 Department of Anesthesiology, Shanxi Bethune Hospital, Shanxi Academy of Medical Sciences, Tongji Shanxi Hospital, Third Hospital of Shanxi Medical University, Taiyuan, 030032 China; 4 Department of Stomatology, Shanxi Bethune Hospital, Shanxi Academy of Medical Sciences, Tongji Shanxi Hospital, Third Hospital of Shanxi Medical University, Taiyuan, 030032 China

**Keywords:** Dental anxiety, Non-pharmacological, Third molar, Bayesian, Virtual reality, Audiovisuals

## Abstract

**Background:**

Tooth extraction is a common procedure performed by oral and maxillofacial surgeons or dentists, often resulting in dental fear and anxiety. The use of relaxing music, audiovisuals, and virtual reality (VR) technologies has been employed to reduce dental anxiety. This network meta-analysis (NMA) aimed to assess the comparative effectiveness of relaxing music, audiovisuals, and VR in reducing dental anxiety associated with tooth extraction.

**Methods:**

Four electronic databases were searched up to March 8, 2023, to identify randomized controlled trials (RCTs) evaluating different multimedia interventions, including the application of using relaxing music, audiovisuals, and VR technologies for dental anxiety. Studies utilizing various anxiety scales for tooth extraction were considered eligible. The pooled standard mean difference (SMD) and 95% confidence interval (CI) of anxiety scale scores were analyzed using Bayesian NMA.

**Results:**

A total of 11 RCTs were included in this NMA. The Bayesian NMA results demonstrated that relaxing music (SMD = -0.64, 95% CI: -1.04, -0.25) and VR (SMD = -0.54, 95% CI: -1.08, -0.02) were associated with a reduction in dental anxiety, while audiovisuals (SMD = -0.34, 95% CI: -0.97, 0.33) required further consideration. Ranking probabilities indicated that relaxing music might be the most acceptable method for individuals with dental anxiety. The frequentist NMA yielded consistent rankings in a sensitivity analysis.

**Conclusions:**

Relaxing music shows the greatest potential for reducing dental anxiety related to tooth extraction when compared to other multimedia interventions.

**Supplementary Information:**

The online version contains supplementary material available at 10.1186/s12903-023-03407-y.

## Introduction

Tooth extraction is a common procedure performed by oral and maxillofacial surgeons or dentists, often leading to dental fear and anxiety. Regardless of the tooth being removed, both children and some adults experience dental anxiety or fear during the extraction surgery. Anxiety levels can be particularly high for the extraction of impacted third molars. Dental anxiety refers to an intense fear of dental-related situations or events, affecting a significant percentage of patients undergoing procedures such as implant surgeries or tooth extractions, with rates ranging from 54 to 92% [1]. Individuals with pre-existing hypertension may even face life-threatening medical emergencies, such as hypertensive and tachycardic emergencies, due to the stress of tooth extraction [2]. Therefore, it is crucial to effectively address the anxiety of these individuals.

The management of dental anxiety involves three main approaches: medication, medical device development, and non-pharmacological interventions. Various medications, including benzodiazepines, barbiturates, nitrous oxide (N_2_O), opioids, and phytotherapeutics, have been suggested for treating dental anxiety [1, 3–7]. Improved dental anesthesia injection techniques and tooth extraction methods can also effectively reduce patients' dental anxiety [8–10]. Additionally, non-pharmacological techniques, such as hypnosis [11–13], massage [14], and the use of relaxing music, audiovisuals, and virtual reality technologies, have been employed to decrease dental anxiety.

In today's society, people often watch movies and listen to music. By momentarily diverting patients' attention during tooth extraction procedures, these interventions may effectively minimize dental fear. Individuals who frequently use virtual reality (VR) technology can enhance their sense of immersion by wearing three-dimensional (3D) glasses. These non-pharmacological interventions are non-invasive, safe, and easily adaptable for every person.

Anxiety and fear are subjective experiences that vary in intensity, severity, and expression among individuals. Therefore, objectively quantifying these experiences is challenging. Consequently, numerous anxiety scales have been developed since 1969 [15], with ongoing efforts to refine them. Previous reviews of dental anxiety and fear scales, conducted by Chi, revealed that each scale approaches dental anxiety and fear with its own criteria and perspectives: some scales focus on the pain caused by dental procedures, others on the patient-dentist relationship, and most on the clinical situations encountered in dentistry [16]. It is important to note that the subjective nature of these measurements can introduce limitations and potential ambiguity in scoring procedures. Therefore, ensuring clarity and consistency in scoring methods is crucial to obtain reliable and valid results. Meta-analysis and network meta-analysis are powerful tools for synthesizing evidence from multiple studies, enhancing precision, and informing clinical decision-making. They help overcome the limitations of individual studies and provide a more robust and comprehensive assessment of treatment effects.

Several randomized controlled trials (RCTs) comparing audiovisuals, music, or virtual reality with conventional clinical standard care have been published and gained increased support from dentists. A systematic review assessing the effectiveness of listening to music for reducing anxiety and pain during third molar extraction revealed that music can effectively reduce preoperative or intraoperative anxiety [17]. However, another systematic analysis found that an informative video about third molar removal operations did not significantly reduce pre- and postoperative dental anxiety [18]. Currently, no direct comparative RCTs or network meta-analyses (NMAs) exist to contrast the therapeutic effects of these interventions and determine which one is most effective in reducing patients' dental anxiety. Therefore, it is necessary to evaluate the direct and indirect effects of these non-pharmacological therapies on reducing dental anxiety and fear during tooth extraction using an NMA in order to assist dentists in deciding whether it is worthwhile to transition from music or audiovisuals to virtual reality technology.

## Methods

This NMA followed the Preferred Reporting Items for System Reviews and Meta-Analyses (PRISMA) 2020 statement and was registered on PROSPERO (CRD42022384989).

### Search strategy

Searches for RCTs of dental anxiety in tooth extraction surgery were conducted in four electronic databases, including PubMed, Embase, Cochrane Library, and Scopus, using relevant keywords, study-type filters, and Boolean logic retrievals: “((tooth extraction) OR (third molar surgery)) AND (anxiety) AND ((virtual reality) OR (audiovisual) OR (video) OR (music) OR (multimedia) OR (audio) OR (song))”. The literature search was carried out separately by two authors. The search was not limited by language or date of publication, and all studies released before March 8, 2023, were included.

### Inclusion criteria

The study selection process followed the PICOS question framework: (P) patients included were children or adults without systemic diseases or regular medication use, requiring tooth extraction (primary teeth or third molars); (I) interventions, including VR equipment (either helmets or 3D eyeglasses), audiovisual/video interventions, and music/song listening (for relaxation purposes, with no restrictions on the types and styles); (C) comparators including clinical standard care, verbal information, traditional behavior guidance procedures, counter-stimulation, tell-show-do, or no special relaxation treatment; (O) outcomes, including dental anxiety assessed using the variable anxiety scales; (S) study design applied to RCTs.

### Excluded criteria

Exclusion criteria were: (1) studies that did not report dental anxiety scales or had unclear outcome indicators or incomplete data; (2) music, audiovisuals, or VR interventions with the informed consent of surgery; (3) the original data included operations other than tooth extraction, such as implant surgery or dental pulp treatment, and it was unable to separate the number of independent tooth extraction participants from the study; (4) binaural beats without melody; and (5) confidential data could not be shared or accessed.

### Data extraction

Two authors independently conducted the electronic searches and selected the studies. Duplicate references were eliminated using EndNote® (Clarivate Analytics®, X9 version) software. Titles and abstracts of the collected citations were screened to identify studies that matched the inclusion criteria. Two authors independently gathered the data from the studies that were included. Conflicts were resolved by discussion between the two authors and consultation with a third author.

### Tools used to assess dental anxiety

Trials were included if they reported at least one anxiety scale. The outcome was a mean change from baseline (before intervention) to the endpoint (after intervention) in dental anxiety. Changed mean differences (MDs) and standard deviations (SDs) were taken from the studies or computed following Chapter 6 of the Cochrane Handbook for Systematic Reviews of Interventions. Where the study only offered a mean bar chart with standard deviation, we tried getting in touch with the corresponding author to request the raw data. For the trials for which the author did not respond, we estimated the mean and standard deviation using Image J software (1.53t, NIH, Bethesda, MD, USA). Studies would combine change scores or post-intervention values only if baseline and post-intervention values were not reported simultaneously. Standard mean differences (SMDs) and 95% confidence intervals (CIs) were used to evaluate effect sizes for continuous variables. Since the studies used various anxiety scales and units of measurement, the effect size measure for the continuous outcome was selected for SMD [19].

### Risk of bias and certainty of the evidence assessment

Two authors independently assessed the risk of bias in the included studies using the RoB 2 tool from the Cochrane Handbook for Systematic Reviews of Interventions [20]. Disputes were resolved through consultation with a third author. The certainty of the evidence was assessed using the Confidence in Network Meta-Analysis (CINeMA) web application [21].

### Strategy for data synthesis

The "gemtc", "rjags", and “dmetar” packages served by R software version 4.2.2 were utilized in this study to show the NMA findings following a hands-on guide [22]. Bayesian NMA was performed using Markov chain Monte Carlo method. The model was created using four Markov chains. The "burn-in" period was set at 5000 simulations for each chain, and the posterior distributions of the model parameters were determined after 100,000 iterations. The geometry of the network was explored using R software and CINeMA web application. SMD and 95% CI were presented for the main outcome. The probability of each intervention was deduced, and the network ranking of the interventions was determined based on the surfaces under the cumulative ranking curve (SUCRA), with higher SUCRA values indicating preferred recommendation. The total consistency in the efficacy network was evaluated using the full design-by-treatment interaction random-effects model and the local consistency was evaluated using the loop-specific and side-splitting approach, with *p* < 0.05 indicating a significant inconsistency existing. Publication bias was assessed using Egger's test when there were more than 10 RCTs available for this meta-analysis, with P < 0.05 indicating the presence of publication bias. Network meta-regression (NMR) analysis was performed to assess the impact of age, anxiety scale, and risk of bias. Using the frequentist framework NMA under the "netmeta" and "dmetar" packages, sensitivity analysis was conducted to evaluate the robustness and reliability of the findings.

## Results

### Studies and treatments

**Fig. 1:**
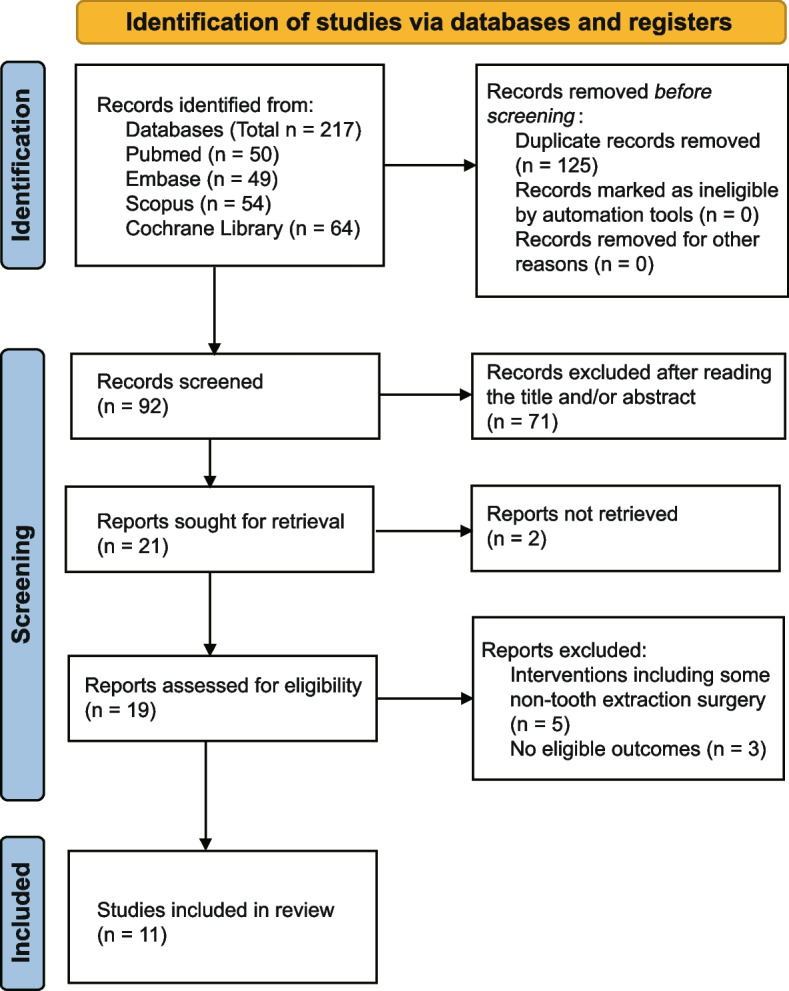
PRISMA flowchart for study selection

**Fig. 2:**
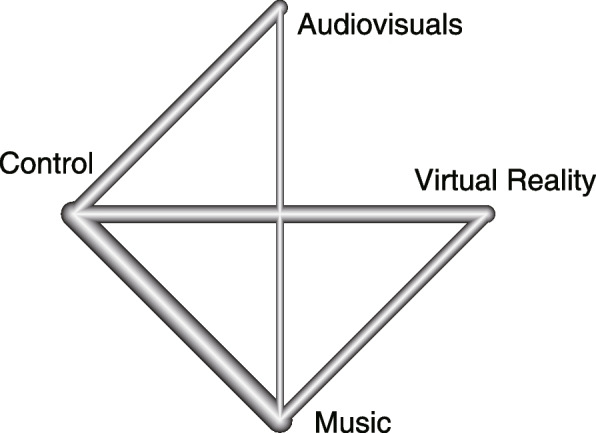
Geometry of the multimedia network for reducing dental anxiety

A total of 217 studies were retrieved. 125 duplicated records were removed, and 71 records were excluded after reading the title and abstract. After reading the full text, 11 RCTs were ultimately included in this review. Search strategy details are shown in Fig. 1 and (Additional file 1). Summarized characteristics of RCTs and participants were demonstrated in Table 1. The studies included were published between 2007 and 2023. There are a total of 8 anxiety scales: the Spielberger State-Trait Anxiety Inventory subscale for trait anxiety (STAI-T), the Spielberger State-Trait Anxiety Inventory subscale for state anxiety (STAI-S), the modified child fear survey schedule dental subscale (CFSS-DS), the visual analog scale (VAS), the modified dental anxiety scale (MDAS), the Corah dental anxiety scale (CDAS), the facial image scale (FIS), and Venham’s Picture Test (VPT). The higher scores on all these scales indicated greater anxiety. The 11 RCTs included 8 two-armed studies [23–30] and 3 three-armed studies [31–33]. Only two studies reported change means and SDs in their papers [27, 29]. In one study with two scales, we got SDs using the 95% CIs for group means [31], and in four studies [23, 25, 26, 30], we imputed SDs for changes from baseline. We had to estimate the SDs by measuring them using Image J software and calculating them from the SE (or SEM, which is almost equal to SE in our estimation) because two articles published means and SDs using bar charts instead of providing the raw data [24, 28]. Only post-intervention values were published in two studies [32, 33], we integrated them into this analysis too. Respect networks are shown in Fig. 2.

**Table 1:** List of included studies (*n* = 11)

Study	Author, year	Intervention	Anxiety scale	Changed mean	Changed SD	n	Population
1	Sorribes De Ramon LA, [31] 2023^a^	CT	STAI-T	-3.53	2.56	91	adult
		MU	STAI-T	-7.74	3.53	91	adult
		VR	STAI-T	-5.83	2.76	93	adult
	Sorribes De Ramon LA, [31] 2023^b^	CT	STAI-S	-3.10	10.51	91	adult
		MU	STAI-S	-8.41	8.54	91	adult
		VR	STAI-S	-3.98	10.01	93	adult
2	Yamashita K, [24] 2022	CT	STAI-S	-3.92	9.04	18	adult
		MU	STAI-S	-12.46	12.29	17	adult
3	Du Q, [23] 2022	CT	CFSS-DS	-1.60	15.51	42	child
		VR	CFSS-DS	-2.26	13.52	41	child
4	Menziletoglu D, [25] 2021	CT	VAS	-0.15	2.99	30	adult
		MU	VAS	-1.77	2.33	30	adult
5	Gs G, [32] 2021*	CT	FIS	3.00	1.00	28	child
		MU	FIS	1.80	0.50	28	child
		VR	FIS	1.50	0.50	28	child
6	Yamashita Y, [27] 2020	CT	VAS	4.00	22.30	49	adult
		VR	VAS	-13.30	28.70	51	adult
7	Luque-Ribas M, [26] 2020^a^	CT	MDAS	-1.90	4.64	15	adult
		AV	MDAS	-2.80	4.36	15	adult
	Luque-Ribas M, [26] 2020^b^	CT	STAI-S	-0.60	4.86	15	adult
		AV	STAI-S	0.30	6.60	15	adult
	Luque-Ribas M, [26] 2020^c^	CT	STAI-T	1.20	7.61	15	adult
		AV	STAI-T	0.60	5.05	15	adult
8	Yamashita K, [28] 2019	CT	STAI-S	-2.80	11.21	17	adult
		MU	STAI-S	-13.13	16.00	17	adult
9	Miyata K, [29] 2016^a^	CT	VAS	2.38	11.38	21	adult (fearful)
		MU	VAS	-11.70	14.90	21	adult (fearful)
	Miyata K, [29] 2016^b^	CT	VAS	0.52	7.04	21	adult (non-fearful)
		MU	VAS	-3.38	6.76	21	adult (non-fearful)
10	Kim YK, [30] 2011	CT	CDAS	0.36	3.00	113	adult
		MU	CDAS	-0.30	3.17	106	adult
11	Prabhakar AR, [33] 2007*	CT	VPT	1.30	1.00	20	child
		MU	VPT	1.70	1.10	20	child
		AV	VPT	1.10	0.90	20	child

Interventions: *CT* Control group, *MU* Music group, *AV* Audiovisuals group, *VR* Virtual reality group

Anxiety scales: *STAI-T* Spielberger State-Trait Anxiety Inventory subscale for trait anxiety, *STAI-S* Spielberger State-Trait Anxiety Inventory subscale for state anxiety, *CFSS-DS* Modified child fear survey schedule dental subscale, *VAS* Visual analog scale, *MDAS* Modified dental anxiety scale, *CDAS* Corah dental anxiety scale, *FIS* Facial image scale, *VPT* Venham’s Picture Test

^*^Studies with post-intervention mean and standard deviation (SD) values. ^a,b,c ^The studies with superscript lowercase letters reported two or three anxiety scales

### Rank probabilities

**Fig. 3:**
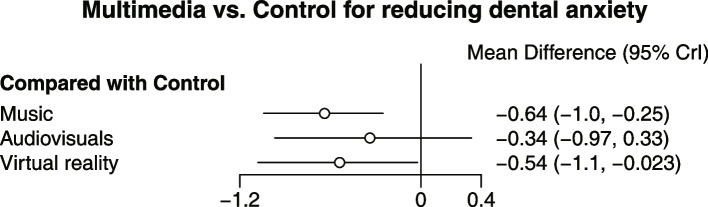
Network forest plot comparing the effectiveness of multimedia in reducing dental anxiety

A total of 11 RCTs including 15 scales were included in the NMA for dental anxiety related to tooth extraction. The NMA results showed that music (SMD = -0.64, 95% CI: -1.04, -0.25) and VR (SMD = -0.54, 95% CI: -1.08, -0.02) interventions were associated with a reduction in dental anxiety compared to control. Pairwise comparisons between the different treatments are depicted in the league table (Table 2). A forest plot of NMA for overall efficacy is shown in Fig. 3. The SUCRA findings revealed the following efficacy ranking: Music > VR > audiovisuals > control with *p* values of 0.82, 0.68, 0.45, and 0.06, respectively. Music had the highest probability of being the most effective intervention in reducing dental anxiety related to tooth extraction, followed by VR. Audiovisual interventions did not show a statistically significant difference (SMD = -0.34, 95% CI: -0.97, 0.33) in reducing dental anxiety compared to control.

**Table 2:** Matrix of pooled results for Bayesian network meta-analysis

	Control	Music	Audiovisuals	Virtual Reality
CT	-	-0.64 (-1.04, -0.25)	-0.34 (-0.97, 0.33)	-0.54 (-1.08, -0.02)
MU	0.64 (0.25, 1.04)	-	0.3 (-0.39, 1.04)	0.1 (-0.47, 0.66)
AV	0.34 (-0.33, 0.97)	-0.3 (-1.04, 0.39)	-	-0.2 (-1.06, 0.59)
VR	0.54 (0.02, 1.08)	-0.1 (-0.66, 0.47)	0.2 (-0.59, 1.06)	-

Effect of intervention in each column compared to intervention in each row

### Risk of bias and certainty of the evidence

**Fig. 4:**
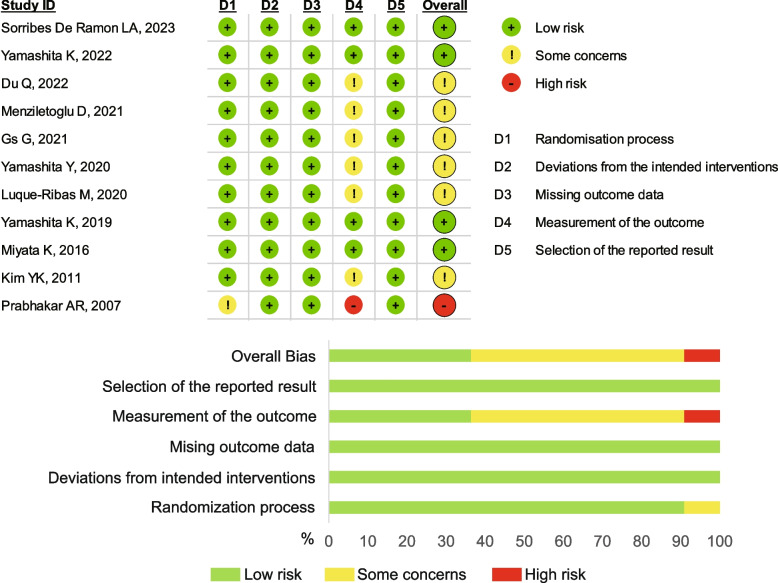
Summary and graph of the risk of bias

**Fig. 5:**
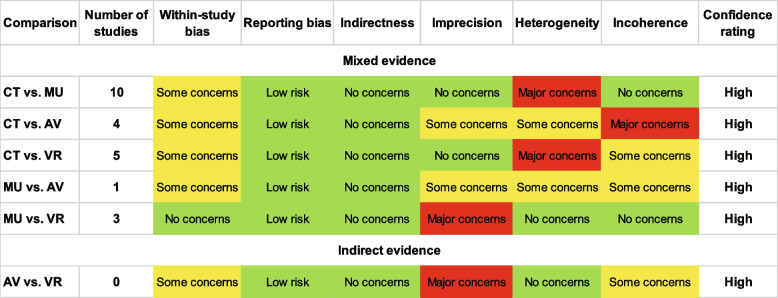
Evaluation of the confidence in the findings from the network meta-analysis (NMA)

Six studies with eight scales received some concerns, four with six scales had a low risk, and one had a high risk. Due to the interventions' obvious nature, participants in such studies could not have been blinded. A summary of the risk of bias assessment for each included study is shown in Fig. 4. The assessment of the evidence's certainty is displayed in Fig. 5. CINeMA rated all of the comparisons for the efficacy outcome as having high confidence in the existing evidence.

### Publication bias

**Fig. 6:**
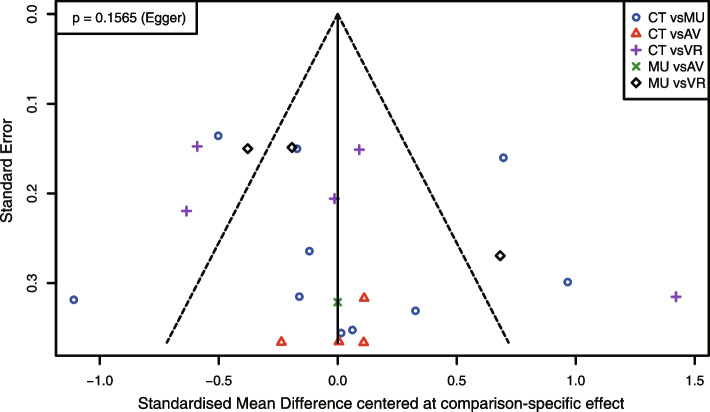
Comparison-adjusted funnel plot for the NMA

The comparison-adjusted funnel plot symmetrically displays the publication bias of NMA for 11 RCTs (Fig. 6). The result of Egger's test was also not statistically significant (*p* = 0.16).

### Transitivity and inconsistency analysis

The full design-by-treatment random effects model did not show significant between-design inconsistency (*p* = 0.07). And the network of the indirect comparison between AV and MU contained statistically significant local inconsistency (*p* = 0.0497). This *p*-value was rounded to 0.05. We believed that inconsistency factors continued to exist even though a result of *p* > 0.05 could be obtained by altering the Markov chain Monte Carlo method's calculation parameters. To evaluate potential causes of inconsistency, an NMR was conducted.

### NMR analysis

**Fig. 7:**
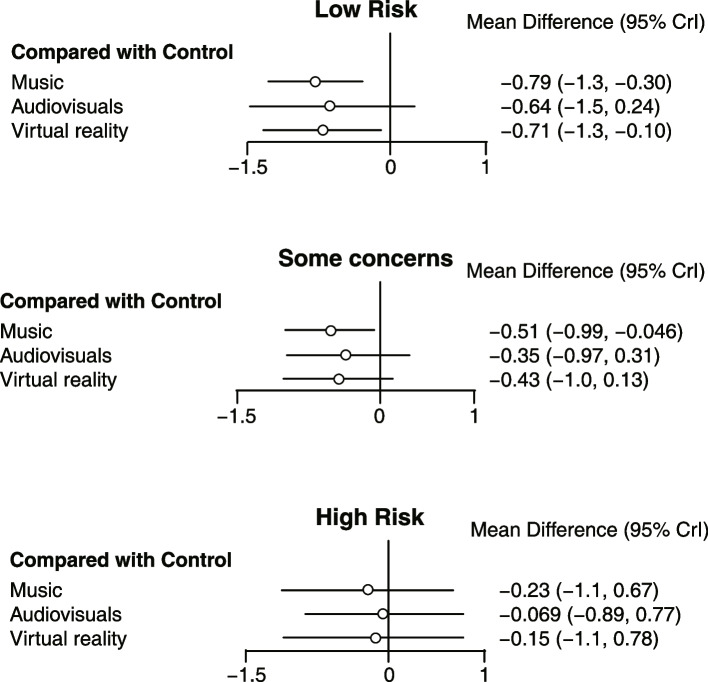
Network meta-regression forest plot controlling for the risk of bias

The NMR analysis examined the impact of risk of bias, anxiety scale, and age on the results. In the RoB variable group, there were three levels: low risk, some concerns, and high risk. The scale group included five levels: STAI-T, STAI-S, DAS, VAS, and others. Child and adult were the two levels in each age group. To determine if the NMR model fitted the data more accurately, the deviance information criteria (DIC) of the subsequent NMR model developed and the previous NMA model was compared. A better match is indicated by lower DIC values. Our NMR model's DIC value (DIC = 28.06) is lower than that of our previous model (DIC = 35.40), which did not control for RoB. However, scale (DIC = 35.51) and age (DIC = 35.12) had almost no effect on the value of DIC. The findings of the NMR showed that RoB contributed to local inconsistency, and the data were more accurately fitted by controlling for RoB than they were without the covariate (Fig. 7). We may conclude that the possibility of bias did not affect the results because the empirical means and 95% CIs did encompass zero. The empirical means and 95% CIs for RoB, scale, and age were 0.36 (-0.34, 1.04), -0.06 (-0.74, 0.61), and 0.61 (-0.57, 0.78), respectively.

### Sensitivity analysis

**Fig. 8:**
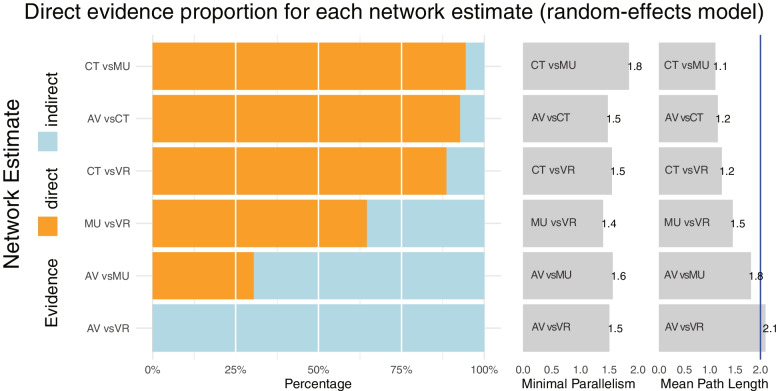
Proportion of direct evidence for each network estimate in the frequentist NMA

**Fig. 9:**
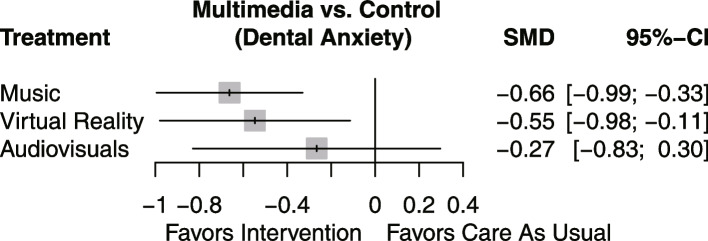
Forest plot of the frequentist NMA comparing different multimedia therapies

**Fig. 10:**
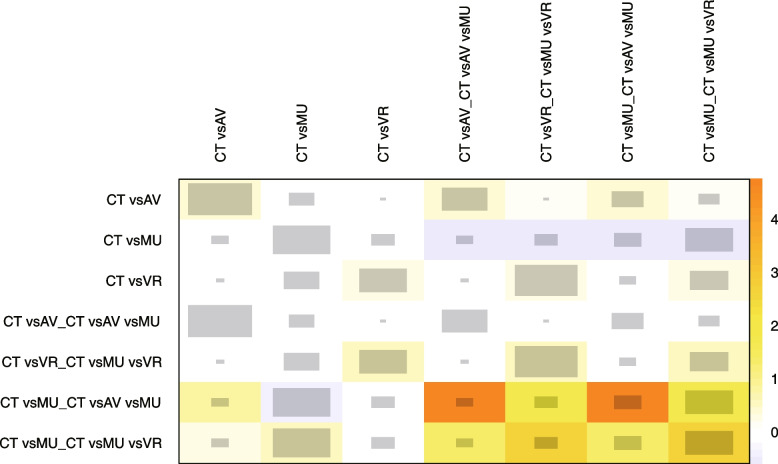
Heatmap of the network designed in rows compared to columns

**Fig. 11:**
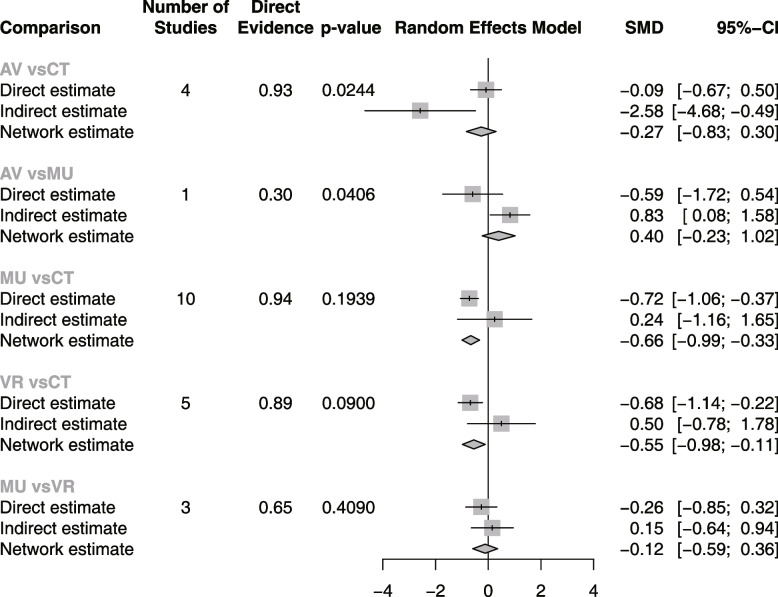
Forest plot of the frequentist NMA comparing the difference between effect estimates based on direct and indirect evidence

We used NMA from the frequentist framework to perform the sensitivity analysis. The frequentist framework has a different conceptual and mathematical foundation from Bayesian NMA. Direct evidence proportion for each network estimate (random-effects model) in frequentist NMA is presented in Fig. 8, with a mean path length > 2 means that a comparison estimate should be interpreted with particular caution. Frequentist NMA forest plot of the different multimedia therapies in reducing the dental anxiety related tooth extraction is presented in Fig. 9. and both Bayesian and frequentist NMA approaches effectively yielded similar results. The net heatmap designed in a row compared to in the column is presented in Fig. 10, with a deep red background indicates strong inconsistency, the bigger the gray box, the more important the comparison. A significant disagreement (inconsistency) between the direct and indirect estimate in (AV vs. CT) and (AV vs. MU) comparisons is presented in Fig. 11. Frequentist network meta-analysis matrix of pooled results is shown in Additional file 2.

## Discussion

Many technological interventions are used to divert attention from unpleasant stimuli. More focus has been placed on non-pharmacological and cost-effective therapies like music and audiovisuals. Innovative, pleasant, and immersive therapies may have gotten more attention. The most common surgical operation in dental clinics is tooth extraction, which puts patients at a higher risk of developing dental anxiety and panic. Some systematic reviews and meta-analyses have shown that music and audiovisuals can reduce dental anxiety [34–36]. However, other studies have raised questions about the potential benefits of informative audiovisuals in lowering dental anxiety [18, 37]. The informative videos used in these trials mostly focused on how to perform tooth surgery, which could be distressing for patients [38]. In this study, RCTs using informative audio or video materials were excluded, as were all dental operations other than a tooth extraction. For tooth extraction-related dental anxiety, only relaxing music, audiovisuals, and virtual reality were included. An NMA was carried out to evaluate the contrasting and ranking of the various therapies.

It is common practice to assess anxiety using various scales, such as the STAI-S, STAI-T, DAS, VAS, and others. The STAI-S assesses transient anxiety and accurately captures the level of anxiety present at the time of the assessment. The STAI-T measures permanent anxiety and reflects the level of anxiety on ordinary occasions. The validated tools CDAS, MDAS, and CFSS-DS are used specifically to measure dental anxiety. Other scales, like VAS, FIS, and VPT are also used to test anxiety scores. These scales have different signaling questions and score estimations. We standardized the outcomes and transformed the mean to SMD to improve precision.

The NMA reviewed and analyzed the majority of available RCT data, providing evidence that the use of multimedia can effectively reduce dental anxiety caused by tooth extraction. With a sample size of over 1000 individuals, we combined direct and indirect evidence from 11 RCTs (15 scales) comparing three different intervention arms with traditional standard care. According to the NMA findings, the top two methods for reducing dental anxiety were music and VR. Surprisingly, music ranked higher in reducing anxiety compared to audiovisuals and VR. These conclusions were supported by evidence of moderate to high quality.

It should be noted that there are limited published network meta-analyses on music, audiovisuals, and virtual reality, so direct comparisons with other network conclusions cannot be made. However, insights can be drawn from conventional meta-analyses that have already been published, as well as systematic reviews.

Music is a low-cost, effective, and commonly used non-pharmacological technique that diverts patients' attention away from stressful stimuli and reduces dental anxiety. Among the studies considered in this NMA, the proportion of evidence supporting a direct comparison between music and conventional treatment (CT) was the highest, followed by audiovisuals (AV) vs. CT, and virtual VR vs. CT, respectively (Fig. 8). These findings align with the previous findings of the pre-reviewers, who found that listening to relaxing music can significantly reduce dental anxiety [17, 39, 40]. However, it is worth noting that earlier research has identified musical factors as a potential hindrance to meta-analysis, as the effects of music can vary depending on genres, frequencies, and rhythms. In this study, researchers or patients themselves selected relaxing music as the primary assessment in each of the included RCTs, which may have contributed to the successful completion of this NMA.

By combining both sounds and images, audiovisuals provide patients with additional audio-visual information and, in theory, should be easier to use as a distraction. However, the findings of the study suggest that music is more effective than audiovisuals in reducing dental anxiety. Several reviewers have also noted that there is limited and weak evidence supporting the use of audiovisuals in reducing dental anxiety [34, 41–43]. Nevertheless, earlier reviews have indicated that audiovisuals can be beneficial for reducing dental anxiety in children [35, 44]. It is important to note that the 95% confidence intervals for both the Bayesian NMA (Fig. 3) and the frequentist NMA (Fig. 9) do not exclude the no-effect line, which calls for caution when concluding that audiovisuals can effectively reduce dental anxiety.

Over the past decade, the use of VR technology in healthcare has rapidly advanced. Compared to other multimedia technologies, VR offers advantages such as haptic feedback and immersive visualization. In our NMA, we found that even though VR devices used in tooth extractions only focus on immersive vision and synchronized sound, they can still significantly reduce dental anxiety. Our assessment of VR aligns with findings from other studies [36, 40]. However, it is worth noting that VR currently ranks lower than music in terms of its potential to alleviate dental anxiety, based on a limited number of studies.

### Limitations

In our findings, music should be prioritized based on its rank probability of effectiveness. If music is ineffective, VR may be considered as an alternative option. However, there are several limitations that may impact our findings. Firstly, the number of studies included in this NMA is limited, while there are numerous musical instruments and audiovisuals with diverse regional and ethnic characteristics. Additionally, different musical instruments and visuals can result in different auditory experiences, which necessitates cautious interpretation of the study's findings. Secondly, NMA relies on data from both direct and indirect comparisons, and the strength of the evidence can be improved with more substantial direct comparisons.

## Conclusion

Based on this NMA, the use of relaxing music may be prioritized as a non-pharmacological intervention to reduce dental anxiety associated with tooth extraction in both adults and children. This conclusion can be valuable for dentists or patients when selecting interventions to alleviate dental anxiety. However, it is important to note that the certainty of the evidence supporting this conclusion is only moderate due to the sample size. To enhance the certainty of this NMA, further large-scale studies with direct comparisons and 3-arm designed RCTs are required.

### Supplementary Information

**Additional file 1: Table 1.** Retrieval strategy (2023/3/8).

**Additional file 2: Table 2.** Frequentist network meta-analysis matrix of pooled results.

## Data Availability

The datasets supporting the conclusions are included in the article and its additional files.

## Abbreviations

RCT: Randomized controlled trial
NMA: Network meta-analysis
PRISMA: Preferred Reporting Items for System Reviews and Meta-Analyses
SMD: Standard mean differences
CI: Confidence interval
CINeMA: Confidence in Network Meta-Analysis
SUCRA: Surfaces under the cumulative ranking curve
NMR: Network meta-regression
STAI-T: Spielberger State-Trait Anxiety Inventory subscale for trait anxiety
STAI-S: Spielberger State-Trait Anxiety Inventory subscale for state anxiety
CFSS-DS: Modified child fear survey schedule dental subscale
VAS: Visual analog scale
MDAS: Modified dental anxiety scale
CDAS: Corah dental anxiety scale
FIS: Facial image scale
VPT: Venham’s Picture Test
DIC: Deviance information criteria

## References

[1] Cai H, Xi P, Zhong L, Chen J, Liang X (2021). Efficacy of aromatherapy on dental anxiety: A systematic review of randomised and quasi-randomised controlled trials. Oral Dis.

[2] Qin Z, Zhou C, Zhu Y, Wang Y, Cao H, Li W, Huang Z (2022). Virtual Reality for Hypertension in Tooth Extraction: A Randomized Trial. J Dent Res.

[3] Kim JY, Park SY, Han YS, Lee H (2022). Comparison of vital sign stability and cost effectiveness between midazolam and dexmedetomidine during third molar extraction under intravenous sedation. J Korean Assoc Oral Maxillofac Surg.

[4] Shafi RI, Goswami M, Rahman B, Nangia T, Nisa TU, Chawla S (2021). Comparative Evaluation of Changes in Physiological and Psychomotor Effects in Pediatric Patients during Extraction under Different Concentrations of Nitrous Oxide-Oxygen Inhalation Sedation. Contemp Clin Dent.

[5] da Cunha RS, Amorim KS, Gercina AC, de Oliveira ACA, Dos Santos Menezes L, Groppo FC, Souza LMA (2021). Herbal medicines as anxiolytics prior to third molar surgical extraction. A randomized controlled clinical trial. Clin Oral Investig.

[6] Arslan I, Aydinoglu S, Karan NB (2020). Can lavender oil inhalation help to overcome dental anxiety and pain in children? A randomized clinical trial. Eur J Pediatr.

[7] Brignardello-Petersen R (2020). There seems to be similar control of anxiety with diazepam, midazolam, and nitrous oxide in patients undergoing surgical maxillary third-molar extraction. J Am Dent Assoc.

[8] Elicherla SR, Bandi S, Nunna M, Saikiran KV, Sahithi V, Nuvvula S (2021). Comparative evaluation of efficacy of Physics Forceps versus conventional forceps in pediatric dental extractions: a prospective randomized study. J Dent Anesth Pain Med.

[9] Hao Y, Zhang Z, Meng Y (2021). Application Effect of Computer-Assisted Local Anesthesia in Patient Operation. Contrast Media Mol Imaging.

[10] Brunton PA, McLean M, Vedagiri S, McKeage J, Ruddy B, Weatherly K, White D, Taberner A, Loch C (2022). Jet injection needle-free dental anaesthesia: Initial findings. J Dent.

[11] Facco E, Bacci C, Zanette G (2021). Hypnosis as sole anesthesia for oral surgery: The egg of Columbus. J Am Dent Assoc.

[12] Sabherwal P, Kalra N, Tyagi R, Khatri A, Srivastava S (2021). Hypnosis and progressive muscle relaxation for anxiolysis and pain control during extraction procedure in 8–12-year-old children: a randomized control trial. Eur Arch Paediatr Dent.

[13] Mackey EF (2010). Effects of hypnosis as an adjunct to intravenous sedation for third molar extraction: a randomized, blind, controlled study. Int J Clin Exp Hypn.

[14] Kunusoth R, Colvenkar S, Alwala AM, Sampreethi S, Ahmed MS (2022). Massage Therapy to Control Anxiety Before Extraction of an Impacted Tooth. Cureus.

[15] Corah NL (1969). Development of a dental anxiety scale. J Dent Res.

[16] Chi SI (2023). What is the gold standard of the dental anxiety scale?. J Dent Anesth Pain Med.

[17] Monteiro J, da Silva Barbirato D, Moraes SLD, Pellizzer EP, do Egito Vasconcelos BC (2022). Does listening to music reduce anxiety and pain in third molar surgery-a systematic review. Clin Oral Investig..

[18] Souza MRF, Gonçalves MWA, de Souza GM, Fernandes IA, Galvão EL, Falci SGM. Does watching an informative video reduce the anxiety in patients undergoing third molar surgery: a systematic review of randomized controlled trials. Oral Maxillofac Surg. 2022; Online ahead of print. 10.1007/s10006-022-01132-4 36525143

[19] Murad MH, Wang Z, Chu H, Lin L (2019). When continuous outcomes are measured using different scales: guide for meta-analysis and interpretation. BMJ.

[20] Sterne JAC, Savovic J, Page MJ, Elbers RG, Blencowe NS, Boutron I, Cates CJ, Cheng HY, Corbett MS, Eldridge SM (2019). RoB 2: a revised tool for assessing risk of bias in randomised trials. BMJ.

[21] Nikolakopoulou A, Higgins JPT, Papakonstantinou T, Chaimani A, Del Giovane C, Egger M, Salanti G (2020). CINeMA: An approach for assessing confidence in the results of a network meta-analysis. PLoS Med.

[22] Harrer M, Cuijpers P, Furukawa T, Ebert D (2021). Doing Meta-Analysis with R: A Hands-On Guide.

[23] Du Q, Ma X, Wang S, Zhou S, Luo C, Tian K, Fei W, Liu X (2022). A digital intervention using virtual reality helmets to reduce dental anxiety of children under local anesthesia and primary teeth extraction: A randomized clinical trial. Brain Behav.

[24] Yamashita K, Uto A, Uchino M, Kibe T, Sugimura M. Listening to music before tooth extraction attenuates sympathetic nervous system activity: A randomized control trial. Oral Sci Int. 2023;20(2):88–94.

[25] Menziletoglu D, Guler AY, Cayir T, Isik BK (2021). Binaural beats or 432 Hz music? which method is more effective for reducing preoperative dental anxiety?. Med Oral Patol Oral Cir Bucal.

[26] Luque-Ribas M, Figueiredo R, Guerra-Pereira I, Valmaseda-Castellón E (2020). Effect of audiovisual eyeglasses on intraoperative pain, anxiety, and hemodynamic changes during mandibular third molar extraction: a randomized controlled clinical trial. Quintessence Int.

[27] Yamashita Y, Shimohira D, Aijima R, Mori K, Danjo A (2020). Clinical Effect of Virtual Reality to Relieve Anxiety During Impacted Mandibular Third Molar Extraction Under Local Anesthesia. J Oral Maxillofac Surg..

[28] Yamashita K, Kibe T, Ohno S, Kohjitani A, Sugimura M (2019). The Effects of Music Listening During Extraction of the Impacted Mandibular Third Molar on the Autonomic Nervous System and Psychological State. J Oral Maxillofac Surg.

[29] Miyata K, Odanaka H, Nitta Y, Shimoji S, Kanehira T, Kawanami M, Fujisawa T (2016). Music before Dental Surgery Suppresses Sympathetic Activity Derived from Preoperative Anxiety: A Randomized Controlled Trial. JDR Clin Trans Res.

[30] Kim YK, Kim SM, Myoung H (2011). Musical intervention reduces patients' anxiety in surgical extraction of an impacted mandibular third molar. J Oral Maxillofac Surg.

[31] Sorribes de Ramon LA, Ferrandez Martinez AF, Garcia Carricondo AR, Espin Galvez F, Alarcon Rodriguez R (2023). Effect of virtual reality and music therapy on anxiety and perioperative pain in surgical extraction of impacted third molars. J Am Dent Assoc.

[32] Gs G, George S, Anandaraj S, Sain S, Jose D, Sreenivas A, Pillai G, Mol N (2021). Comparative Evaluation of the Efficacy of Virtual Reality Distraction, Audio Distraction and Tell-show-do Techniques in Reducing the Anxiety Level of Pediatric Dental Patients: An In Vivo Study. Int J Clin Pediatr Dent.

[33] Prabhakar AR, Marwah N, Raju OS (2007). A comparison between audio and audiovisual distraction techniques in managing anxious pediatric dental patients. J Indian Soc Pedod Prev Dent.

[34] Lu C, Zhang YY, Xiang B, Peng SM, Gu M, Wong HM (2023). Management of fear and anxiety in dental treatments: a systematic review and meta-analysis of randomized controlled trials. Odontology.

[35] Zhang C, Qin D, Shen L, Ji P, Wang J (2019). Does audiovisual distraction reduce dental anxiety in children under local anesthesia? A systematic review and meta-analysis. Oral Dis.

[36] Gurav KM, Kulkarni N, Shetty V, Vinay V, Borade P, Ghadge S, Bhor K (2022). Effectiveness of Audio and Audio-Visual Distraction Aids for Management of Pain and Anxiety in Children and Adults Undergoing Dental Treatment- A Systematic Review And Meta-Analysis. J Clin Pediatr Dent.

[37] Astramskaite I, Poskevicius L, Juodzbalys G (2016). Factors determining tooth extraction anxiety and fear in adult dental patients: a systematic review. Int J Oral Maxillofac Surg.

[38] Toledano-Serrabona J, Sánchez-Torres A, Camps-Font O, Figueiredo R, Gay-Escoda C, Valmaseda-Castellón E (2020). Effect of an Informative Video on Anxiety and Hemodynamic Parameters in Patients Requiring Mandibular Third Molar Extraction: A Randomized Clinical Trial. J Oral Maxillofac Surg.

[39] Bradt J, Teague A (2018). Music interventions for dental anxiety. Oral Dis.

[40] Hoffmann B, Erwood K, Ncomanzi S, Fischer V, O'Brien D, Lee A (2022). Management strategies for adult patients with dental anxiety in the dental clinic: a systematic review. Aust Dent J.

[41] Prado IM, Carcavalli L, Abreu LG, Serra-Negra JM, Paiva SM, Martins CC (2019). Use of distraction techniques for the management of anxiety and fear in paediatric dental practice: A systematic review of randomized controlled trials. Int J Paediatr Dent.

[42] Liu Y, Gu Z, Wang Y, Wu Q, Chen V, Xu X, Zhou X (2019). Effect of audiovisual distraction on the management of dental anxiety in children: A systematic review. Int J Paediatr Dent.

[43] De Stefano R, Bruno A, Muscatello MR, Cedro C, Cervino G, Fiorillo L (2019). Fear and anxiety managing methods during dental treatments: a systematic review of recent data. Minerva Stomatol.

[44] Quek JS, Lai B, Yap AU, Hu S (2022). Non-pharmacological management of dental fear and anxiety in children and adolescents: An umbrella review. Eur J Paediatr Dent.

