# Supplementary material for: A systematic review and network meta-analysis of virtual reality, audiovisuals and music interventions for reducing dental anxiety related to tooth extraction
Source: BMC Oral Health. 2023 Sep 22;23:684. doi: 10.1186/s12903-023-03407-y (PMC10515077; doi:10.1186/s12903-023-03407-y)
Supplement: Supplementary file 1 — Additional file 1: Table 1. Retrieval strategy (2023/3/8). [file 12903_2023_3407_MOESM1_ESM.docx]

| **Additional file 1: Table S1.** Retrieval strategy (2023/3/8) | |
| --- | --- |
| **Database** | **Search terms** |
| **Pubmed** | |
| #1 | ((tooth extraction) OR (third molar surgery)) AND (anxiety) AND ((virtual reality) OR (audiovisual) OR (video) OR (music) OR (multimedia) OR (audio) OR (song)) |
| Results | 50 |
| **Embase** | |
| #1 | ('tooth extraction'/exp OR 'tooth extraction' OR (('tooth'/exp OR tooth) AND ('extraction'/exp OR extraction)) OR 'third molar surgery'/exp OR 'third molar surgery' OR (third AND ('molar'/exp OR molar) AND ('surgery'/exp OR surgery))) AND ('anxiety'/exp OR anxiety) AND ('virtual reality'/exp OR 'virtual reality' OR (virtual AND ('reality'/exp OR reality)) OR audiovisual OR 'video'/exp OR video OR 'music'/exp OR music OR 'multimedia'/exp OR multimedia OR audio OR 'song'/exp OR song) |
| Results | 49 |
| **Scopus** | |
| #1 | TITLE-ABS-KEY ( ( ( tooth AND extraction ) OR ( third AND molar AND surgery ) ) AND ( anxiety ) AND ( ( virtual AND reality ) OR ( audiovisual ) OR ( video ) OR ( music ) OR ( multimedia ) OR ( audio ) OR ( song ) ) ) |
| Results | 54 |
| **Cochrane library** | |
| #1 | ((tooth extraction) OR (third molar surgery)) AND (anxiety) AND ((virtual reality) OR (audiovisual) OR (video) OR (music) OR (multimedia) OR (audio) OR (song)) in All Text |
| Results | 64 |
