# Supplementary material for: A systematic review and network meta-analysis of virtual reality, audiovisuals and music interventions for reducing dental anxiety related to tooth extraction
Source: BMC Oral Health. 2023 Sep 22;23:684. doi: 10.1186/s12903-023-03407-y (PMC10515077; doi:10.1186/s12903-023-03407-y)
Supplement: Supplementary file 2 — Additional file 2: Table 2. Frequentist network meta-analysis matrix of pooled results. [file 12903_2023_3407_MOESM2_ESM.docx]

**Additional file 2: Table S2.** Matrix of pooled results for frequentist network meta-analysis

|  | Control | Music | Audiovisuals | Virtual reality |
| --- | --- | --- | --- | --- |
| CT | - | -0.66 (-0.99, -0.33) | -0.27 (-0.83, 0.30) | -0.55 (-0.98, -0.11) |
| MU | 0.66 (0.33, 0.99) | - | 0.40 (-0.23, 1.02) | 0.12 (-0.36, 0.59) |
| AV | 0.27 (-0.30, 0.83) | -0.40 (-0.12, 0.23) | - | -0.28 (-0.98, 0.42) |
| VR | 0.55 (0.11, 0.98) | -0.12 (-0.59, 0.36) | 0.28 (-0.42, 0.98) | - |

Note: Effect of intervention in each column compared to intervention in each row.

­­
